# Supplementary material for: “The worst thing is lying in bed thinking ‘I want a cigarette’” a qualitative exploration of smoker’s and ex-smoker’s perceptions of sleep during a quit attempt and the use of cognitive behavioural therapy for insomnia to aid cessation
Source: PLoS One. 2024 May 8;19(5):e0299702. doi: 10.1371/journal.pone.0299702 (PMC11078348; doi:10.1371/journal.pone.0299702)
Supplement: S1 File — (PDF) [file pone.0299702.s001.pdf]

“The worst thing is lying in bed thinking ‘I want a cigarette’” Exploration of smoker’s and ex-smoker’s perceptions of sleep during a quit attempt and the use of cognitive behavioural therapy for insomnia to aid cessation.

Joe A. Matthews, Victoria R. Carlisle, Robert Walker, Emma J. Dennie, Claire Durant, Ryan McConville, Hanna K. Isotalus, and Angela S. Attwood

Supplementary File: Additional information

Research team and reflexivity

Joe Matthews MSc is an experienced male mixed method researcher who was a PhD student at the University of Bristol. He has worked on multiple qualitative studies relating to behaviour change and health. Joe led data collection and analysis.

Robert Walker PhD is an experienced male qualitative researcher who was working as a Research Associate at the University of Bristol. He has designed and led multiple qualitative studies related to physical activity behaviour in diverse populations, including military veterans. Robert consulted on analysis and manuscript write up.

Vicky Carlisle PhD is an experienced female qualitative researcher who was working as a Senior Research Associate at the University of Bristol. She has designed and led multiple qualitative studies related to drug use, addiction and health. Vicky took part in analysis and consulted on manuscript write up.

Emma Dennie MSc is a female who was working as a Research Associate in the Tobacco and Alcohol Research Group at the University of Bristol. She has an undergraduate and postgraduate degree in Psychology. Emma contributed to analysis.

Interview Guide

Welcome them.

Thank you for agreeing to take part in this interview, your views and opinions are really important to us. Today, we are going to be talking about your experience of sleep both now and in relation to a quit attempt. I will also show you some materials of traditional cognitive behavioural therapy for insomnia. We are doing this to improve future support for quitting smokers.

We are really interested in your honest opinions, we are not here to judge you, and we do not want you to feel like you should answer any of the questions in a certain way, there are no right or wrong answers, and as much detail you can give on the topics as possible is really appreciated. First of all, I've just sent across a link that includes an information sheet, a consent form and then a short survey. Please feel free to ask any questions while you complete these.

- Before we get started, I'd like to tell you that I will be **recording the conversation** to help us remember what you said.
- You can ask for the recording to be stopped at any time.
- After we have written a report about all the opinions we have heard from participants, the recordings will be destroyed.
- We will also change any names or identifying information so none of the information that is written down and recorded can be connected to you in any way.
- Please remember that we can stop the recording at anytime.
- If you do not want to answer a question please say so.
- Have you completed the consent form emailed to you?
- Are you happy to continue?

PID:________________ Initials:______________ Date:_____________

Section 1- Current Sleep Behaviour			
**Check**	**Questions**	**Prompts**	**Notes**
	- How would you describe your sleep ?<br>- In your experience what affects your sleep, this could be in a positive or negative way?<br>- Do you think your smoking affects your sleep? This could be in a positive or negative way.<br>- Do you wake up in the night to smoke?<br>- What do you normally do within the hour before you go to bed?	- Think about how long it takes you to get to sleep, how frequently you wake up at night, how you feel on waking in the morning (tired/energetic)?<br>- TV, phone use, caffeine, alcohol etc.	

Section 2- Sleep and quit attempt.			
**Check**	**Interview Question**	**Prompts**	**Theme emerged?**
	- During your previous quit attempts how has your sleep been? Has it been effected in anyway?<br><br>If yes to above question:<br><br>- How was your sleep quality affected?<br><br>- How did it make you feel?<br><br>- [If sleep was impacted negatively] Did you do anything to try and improve your sleep? If so what did you do? Was it effective?<br><br>- Do you think quality of sleep could affect your ability to resist smoking during a quit attempt?<br><br>- Do you think previous quit attempts you have made have been impacted by the quality of your sleep?<br><br>If yes to above question:<br><br>- How was your quit attempt affected by this?	- Think about if you felt fatigued (Physically, emotionally) or experience daytime drowsiness?<br><br>- Think of any sleep medication you may have used.	

Section3: CBT-I materials			
**Check**	**Questions**	**Prompts**	**Themes emerged?**
	**Introduction to section:**<br><br>*"Research suggests that smoking negatively impacts your sleep. This can be further exacerbated during a quit attempt and make it less likely to have a successful quit attempt.*<br><br>*There are existing treatments that may help improve sleep and we would like to get your thoughts and opinions on these.*<br><br>*To do this I'm going to show you a short video that describes different elements of an existing intervention. After the video I'm going to ask for your thoughts and opinions about each element of the intervention. Does that make sense?"*		

Section3: CBT-I materials			
**Check**	**Questions**	**Prompts**	**Themes emerged?**
	- After seeing the video about CBT-I, if you were to try making some of the changes Theo made, which do you think would be easiest/hardest for you to do? Why?<br>- Which of these recommendations are the least feasible changes to make during a quit attempt? Why?<br>- Is there anything that could be done to make those changes easier?	- Think of potential barriers you may face?<br>- Are there any other components that you think would be hard to for you to do?<br>- Think about what quit attempt specific barriers would you face?<br>- Are these barriers to engagement personal to you or do you think other smokers would struggle?	

Section3: CBT-I materials			
**Check**	**Questions**	**Prompts**	**Themes emerged?**
	- Say you decided to quit smoking. Would you consider adopting CBT-I to improve your sleep? Why?<br><br>- If you decided you were going to quit smoking and try some CBT-I techniques alongside to help with sleep, would you like to start these before your quit attempt or after?<br><br>- Looking at the recommendations on screen, what changes or additions would you make in the context of a quit attempt, if any? Why?<br><br>- On screen you should see each CBT-I component we have been through today. Please rank in order which CBT-I components you are more likely to engage in during a quit attempt? Top being most likely, bottom being least likely.	- Better to start before, on or after quit date?<br><br>***Show snapshots of each component on screen ***<br><br>- Think about if it would be better to start before, on or after quit date?<br><br>- Talk me through why you are ranking each in that order.	

**Section3: CBT-I materials**<br><br>**Delivery**			
**Check**	**Questions**	**Prompts**	**Themes emerged?**
	- How would you like CBT-I to be delivered to you?<br><br>- How do you imagine this being delivered digitally?	- What influences this ? impact on quit attempt?<br><br>Face to face, In a group, digitally on a smartphone or online?<br><br>- Smartphone application, website, smart watch?	

CLOSING (2-3 minutes)

- Is there anything else you'd like to tell us about the things we talked about today?
- Do you have any questions for me?
- We appreciate you sharing your thoughts and opinions with us!

Interviews: Follow-up responses which are neutral.

To end of a point before moving on to the next:

'That's interesting, thank you'

'Thank you for that information'

'Ok, I understand, thank you'

'That is useful to know'

'Thank you for that. Let's move on to the next section'

'I see, that is very useful information. So moving on, can we talk about…..'

To probe/prompt a point:

'Why do you think that is?'

'Could you give me an example?'

'What did you try?'

'Can you expand on that?'

'That's interesting, can you tell me some more/explain that in more detail?'

'Is that always the case?'
