# Supplementary material for: “The worst thing is lying in bed thinking ‘I want a cigarette’” a qualitative exploration of smoker’s and ex-smoker’s perceptions of sleep during a quit attempt and the use of cognitive behavioural therapy for insomnia to aid cessation
Source: PLoS One. 2024 May 8;19(5):e0299702. doi: 10.1371/journal.pone.0299702 (PMC11078348; doi:10.1371/journal.pone.0299702)
Supplement: S1 Table — (PDF) [file pone.0299702.s002.pdf]

“The worst thing is lying in bed thinking ‘I want a cigarette’” Exploration of smoker’s and ex-smoker’s perceptions of sleep during a quit attempt and the use of cognitive behavioural therapy for insomnia to aid cessation.

Joe A. Matthews, Victoria R. Carlisle, Robert Walker, Emma J. Dennie, Claire Durant, Ryan McConville, Hanna K. Isotalus, and Angela S. Attwood

Supplementary File (S2 Table): This is a table of complete participant demographic information

Participant	Age	Sex	Ethnicity	Education	Cigarettes per day	Previous quit attempts	Medication during quit attempt	Readiness to quit ladder	FTND	PSQI
001	31	Female	White British	GCSE / O Level grade A*-C	10	One	Champix (varenicline)	I often think about quitting smoking, but have no plans to quit.	Low to moderately dependent smoker	7
002	25	Male	Black British	Higher education	7	Two	Bupropion	I still smoke, but I have begun to change, like cutting back on the number of cigarettes I smoke. I am ready to set a quit date.	Low to moderately dependent smoker	16
003	25	Male	Black British	A levels	6	Two	Nicotine Replacement (Gum, Lozenge, patches etc)	I definitely plan to quit smoking in the next 6 months.	Moderately dependent smoker	14
004	43	Female	White British	A levels	15	Four	Nicotine Replacement (Gum, Lozenge, patches etc)	I often think about quitting smoking, but have no plans to quit.	Moderately dependent smoker	8
005	28	Female	White British	Higher education	12	Three	E-cigarette	I often think about quitting smoking, but have no plans to quit.	Low to moderately dependent smoker	8
006	25	Female	White British	Higher education	N/A	N/A	E-cigarette	I have quit smoking, but I still worry about slipping back, so I need to keep working on living smoke free.	Ex-smoker	8
007	60	Male	White British	GCSE / O Level grade A*-C	15	Five or more	Champix (varenicline)	I definitely plan to quit smoking in the next 30 days.	Moderately dependent smoker	5
008	43	Female	White British	GCSE / O Level grade A*-C	10	Five or more	N/A	I definitely plan to quit smoking in the next 6 months.	Low to moderately dependent smoker	5

009	28	Male	White British	Higher education	12	Two	E-cigarette	I often think about quitting smoking, but have no plans to quit.	Low dependent smoker	4
010	28	Female	Mixed Other	Higher education	8	Five or more	N/A	I definitely plan to quit smoking in the next 6 months.	Low dependent smoker	6
011	29	Male	White British	Higher education	N/A	N/A	E-cigarette	I have quit smoking, but I still worry about slipping back, so I need to keep working on living smoke free.	Ex-smoker	5
012	46	Female	White Other	Higher education	30	Three	Nicotine Replacement (Gum, Lozenge, patches etc)	I often think about quitting smoking, but have no plans to quit.	Low to moderately dependent smoker	12
013	26	Female	Mixed Other	Higher education	10	Three	N/A	I often think about quitting smoking, but have no plans to quit.	Low to moderately dependent smoker	11
014	31	Male	White British	Higher education	12	Two	E-cigarette	I often think about quitting smoking, but have no plans to quit.	Low to moderately dependent smoker	4
015	22	Male	White British	Qualifications at level 1 and below	10	Two	Nicotine Replacement (Gum, Lozenge, patches etc)	I still smoke, but I have begun to change, like cutting back on the number of cigarettes I smoke. I am ready to set a quit date.	Low to moderately dependent smoker	8
016	57	Male	White British	GCSE / O Level grade A*-C	30	Four	N/A	I often think about quitting smoking, but have no plans to quit.	Highly dependent smoker	7
017	62	Female	White British	Higher education	20	Three	E-cigarette	I definitely plan to quit smoking in the next 6 months.	Moderately dependent smoker	5
